# Social dominance and multiple dimensions of psychopathology: An experimental test of reactivity to leadership and subordinate roles

**DOI:** 10.1371/journal.pone.0250099

**Published:** 2021-04-28

**Authors:** Sheri L. Johnson, Benjamin Swerdlow, Jordan A. Tharp, Serena Chen, Jennifer Tackett, Jamie Zeitzer

**Affiliations:** 1 Department of Psychology, University of California Berkeley, Berkeley, CA, United States of America; 2 Department of Psychology, Northwestern University, Evanston, IL, United States of America; 3 Department of Psychiatry, Stanford University, Stanford, CA, United States of America; University of South Florida, UNITED STATES

## Abstract

**Background:**

Theory and research suggest that social dominance is important for multiple forms of psychopathology, and yet few studies have considered multiple dimensions of psychopathology simultaneously, and relatively few have used well-validated behavioral indices.

**Method:**

Among 81 undergraduates, we used a well-validated experimental approach of assigning participants to a leadership or subordinate position, and we examined how self-rated severity of depression, social anxiety, manic tendencies, and psychopathy relate to psychophysiological and affective reactivity to this role.

**Results:**

Consistent with hypotheses, manic symptoms related to more discomfort in the subordinate role compared to the leadership role, as evidenced by more decline in positive affect, more discomfort, and a larger RSA decline, while depression symptoms related to a more positive response to the subordinate role than the leadership role, including more positive affect and more comfort in the assigned role. Social anxiety was related to discomfort regardless of the assigned role, and those with higher psychopathy symptoms did not show differential response to assigned roles.

**Limitations:**

Findings are limited by the mild symptom levels and absence of hormonal data.

**Conclusions:**

Findings provide novel transdiagnostic evidence for the importance of social dominance to differentiate diverse forms of psychopathology.

## Introduction

Organisms who live in groups naturally create and maintain social hierarchies [1, 2]. The adaptive, biologically-based processes involved in hierarchy have been a rich focus of work across anthropology, sociology, ethology, management, and psychology [3]. These literatures have differentiated multiple dimensions involved in the maintenance and effects of hierarchy, including the desire for social rank (dominance motivation), the behavioral strategies used to pursue social rank, behavioral expressions of dominance and subordination, and social rank (which is sometimes referred to as power). Individuals differ profoundly on their experiences and preferences of each dimension [4].

Each dimension has been carefully studied. Dominance motivations have been shown to have clear physiological, affective, cognitive, and interpersonal correlates [3]. The behavioral strategies involved in the pursuit of social rank can be prosocial or coercive [5]: one route to attaining social rank is to attain prestige through the demonstration of competence and expertise, whereas a different path is to use force and intimidation [6, 7]. Of course, some individuals use both types of strategies [5].

Dynamic increases in social rank have been shown to influence many dimensions that are core to psychopathologies, including emotions such as pride [8], social and emotional sensitivity [9], morality [10], approach motivation, and behavioral inhibition [11–13]. Conversely, lower social rank has been tied to variables of import to depression and anxiety, such as shame [14] and sensitivity to social threats [15], as well as lowered performance on measures of working memory and cognitive inhibition [16]. Here, then, we focus on links of psychopathologies with reactivity to changes in social rank, as assessed by experimentally assigning individuals to leadership and subordination roles.

Multiple syndromes have been conceptualized as the outcome of extremes within the social dominance system. Indeed, narcissism is virtually defined by extremes of social dominance dimensions, with symptom measures designed to capture leadership/authority and superiority/arrogance as core facets [17]. Here, we focus on psychopathologies that may have less overt symptom overlap with the dominance behavioral system. We focus on three affective syndromes—mania, depression, and social anxiety, as well as one facet of psychopathy. Evidence that the dominance system is closely tied to these psychopathology syndromes has accrued from social, psychological, and biological paradigms; from human and non-human animal research; from self-report, observational, and biological methods; and from naturalistic and experimental paradigms [18]. In considering psychopathology, much of the literature focuses on extremes of various social dominance-related dimensions, assessed within individuals. We argue, though, that whether an individual’s preferences match their social context is key, as naturalistic and experimental work indicates that the mismatch between dominance motivation and cues of social rank can trigger poor outcomes, including negative affect and impair task performance [19]. Accordingly, we consider how psychopathology relates to individual differences in social dominance variables, and where evidence is available, to responses to social dominance cues.

Perceptions of higher social rank has been related to higher behavioral approach motivation [20], and relatedly, increased activation [21], heightened confidence, greater desire to attain one’s aims, and willingness to work harder in the pursuit of goals [13], each of which has been related to manic vulnerability [22]. Price [23] noted strong parallels between the behavior of “alpha” animals and manic symptoms. Indeed, many manic symptoms, including grandiosity, talkativeness, goal pursuit, heightened sexuality, and aggression, resemble behavioral concomitants of high power [20]. Empirically, manic symptoms correlate with higher observer and self-ratings of dominant behavior [24]. Outside of acute episodes, manic vulnerability, as measured by the Hypomanic Personality Scale (HPS), is associated with higher explicit and implicit dominance motivation, self-rated power, agentic extraversion [25, 26], willingness to prioritize the pursuit of power over social costs [27], and observer-rated dominant behavior [25, 28] Consistent with the importance of attending to individual’s profiles of dominance within a social context, HPS scores have also been tied to insensitivity to cues of social rank. In one experimental study, manic vulnerability related to a tendency to sustain expansive posture without regard to others’ dominant or subordinate displays [29].

Price [23] theorized that psychopathy was tied to a heightened tendency to seek power. Consistent with this idea, in the triarchic model, boldness (defined by confidence, social assertiveness, emotional resiliency, and venturesome-ness) is one of the core facets of psychopathy [30]. Indeed, multiple indices of psychopathy have been found to correlate with observer- and self-ratings of dominant behavior [26, 31–33] and self-ratings of hubristic pride [34], with links observed in both community and incarcerated populations [35]. Again highlighting the importance of social context, psychopathy also appears related to greater reactivity to cues of social rank, as evidenced by heightened anger in response to threats to power, such as insults, disrespect and commands [36].

Some research suggests that one specific facet of psychopathy–boldness, which has been assessed using Fearless Dominance scales—may be more relevant to the dominance system. For example, self-rated dominance motivation appears particularly correlated with the Fearless Dominance subscale of the Psychopathic Personality Inventory [37]. A similar profile has been observed for behavioral indices. Whereas it is normative to take a complementary subordinate role when interacting with someone who expresses dominance, researchers in one study showed that those with psychopathy increased their dominance behavior in response to an interviewer who acted dominantly, but not in response to an interviewer who acted subordinately). Of note, these effects were specifically related to the Fearless Dominance subscale, and not to other facets of psychopathy [38]. Given the specificity of dominance effects to this facet, we focus on Fearless Dominance here.

While we focus on the Fearless Dominance subscale, we acknowledge that the centrality of Fearless dominance/boldness to psychopathy is a topic of debate because many psychopathic individuals do not display heightened dominance motivation and behavior, and the correlations of boldness with other facets of psychopathy are low [39, 40]. Boldness, though, was part of early conceptualizations of psychopathy of the disorder [e.g., 41] and is associated with expert and lay perceptions of psychopathy [42, 43]. To provide insight into the overall links of psychopathy with dominance, we provide correlations of other psychopathy subscales in S1 File.

Depression has been hypothesized to be triggered by inescapable experiences of subordination [44]. Although submissive behavior is normative in such contexts, submissiveness typically would be expected to cease once a new, stable social rank system is achieved. According to this theory, depression is differentiated by prolonged submissive behavior in response to subordination. Empirically, depression is related to feelings of subordination, inferiority, and shame; self-rated and other-rated submissive behavior; lack of pride; and childhood experiences of subordination in clinical and nonclinical samples [18, 45].

Multiple theories have linked social anxiety to hypersensitivity to cues of social rank and hyper-focus on social comparison [46–48]. It has been argued that individuals with higher social anxiety use subordinate behaviors to protect against conflict with higher rank individuals [49]. Many studies indicate that social anxiety is related to self-rated feelings of subordination and shame, to self-rated and implicit measures of low social rank, and to tendencies to engage in submissive behavior [18, 50].

The social anxiety field is unique in the rich array of behavioral measures used to document dominance behavior [51], including observer [52, 53] and peer-rated submissive behavior [54], as well as acoustic properties of speech that connote submissiveness [50, 55]. Across experimental studies, social anxiety is tied to greater reactivity to cues of social rank or competition. For example, social anxiety symptoms have been related to more “body collapse” during interpersonal competition [56], more vocal signals of submissiveness when placed in a dominant role [57], greater testosterone decline after losing a social competition [58], and heightened heart rate reactivity to dominance cues [59].

In sum, extensive evidence suggests that mania and Fearless Dominance are related to heightened dominance motivation and behavior, whereas depression and social anxiety are related to subordination and submissiveness. Despite the parallels in profiles across syndromes, these literatures are fragmented. Most studies of the dominance system have examined psychopathology syndromes in isolation. Available findings suggest that social anxiety remains robustly tied to subordinate behavior, inferiority, shame, and diminished authentic pride when controlling for depression [18, 26, 60]. Conversely, multiple studies have suggested that controlling for anxiety diminishes links of depression with unassertiveness [18, 60], although effects for shame [1] and self-rated power [27] remain significant. Less is known about overlap of Fearless Dominance and mania-relevant profiles. In addition, different paradigms have been used across syndromes.

### Goals of this study

Previous research in psychopathology has all too often focused on social dominance attitudes, beliefs, and emotions within the individual, but dominance is clearly a social construct, and we aimed to understand how multiple psychopathology syndromes guide response to social cues denoting power or subordination, using a well-validated behavioral approach. Participants were assigned to a leadership or subordinate role while completing a dyadic task [19]. We hypothesized that manic tendencies and Fearless Dominance would be tied to more discomfort in a subordinate role and that depression and anxiety would be tied to greater discomfort in a leadership as compared to subordinate role. In a multi-modal approach to measuring reactivity to complementary vs mismatched roles, we assessed self-rated comfort and affect and psychophysiological reactivity. We expected parallel effects across these channels, as we had no a priori reason to believe that one channel would be more sensitive to the experimental manipulation than another would be. This study is unique in providing a transdiagnostic comparison, experimental manipulation of leadership and subordinate roles, and multimodal measurement.

## Method

### Participants

Undergraduates ages 18 and older and fluent in English (*N* = 948) from a large public university completed online baseline questionnaires for partial course credit. To improve ability to assess individuals with higher levels of psychopathology, 450 participants were emailed an invitation to sign up for the in-person session if they scored in the top 25^th^ percentile of the Psychopathic Personality Inventory, the Hypomanic Personality Scale, or the Social Anxiety Interaction Scale, or endorsed five symptoms persisting at least 2 weeks on the Inventory to Diagnose Depression Lifetime. Others were recruited without regard to scores through a general sign-up page. Two participants were excluded from completing the in-person laboratory session because they incorrectly answered attention check items in the online questionnaires (e.g., “choose 2 for your answer to this item”), leaving a final *N* = 81 (64% female, mean age of 19.76, *SD* = 1.59). Self-reported ethnicity was 41% Asian-American, 30% Caucasian, 16% Latino/Hispanic, 9% Middle Eastern, and 4% other or not identified.

### Measures

Participants completed self-rated psychopathology measures online along with other measures not reported here. All psychopathology measures were chosen for their validity, including robust correlations with diagnostic and symptom severity measures [61–68].

#### Hypomanic Personality Scale (HPS)

The HPS is a 48-item questionnaire to identify risk for the development of manic and hypomanic symptoms [62]. Items cover lifetime experiences of positive affectivity, lack of sleep, racing thoughts, and fast speech. Although the original scale used a true/false format, we used a well-validated response format from 0 = “strongly disagree” to 3 = “strongly agree” to enhance variability [cf. 69]. HPS scores are the average of items (11 reverse-keyed). No participants exceeded the clinical threshold of 36 [62].

#### 7-Up 7-Down

The 7-Up 7-Down is a 14-item short form of the General Behavior Inventory [70] assessing symptoms of mania (7 items) and depression (7 items [68]. Items are rated on a scale ranging from 1 = “never or hardly ever” to 4 = “very often or almost constantly.” Scores for both subscales are the sum of corresponding items.

#### Inventory to Diagnose Depression-Lifetime (IDD-L)

The IDD-L assesses severity of lifetime depressive symptoms [71]. The scale consists of 22 items covering the nine DSM criteria for major depressive disorder (e.g., hopelessness, loss of interest). Ratings were made on a scale ranging from no endorsement (0) to full endorsement (i.e. “I felt tired or exhausted almost all of the time” = 4). For each item endorsed, participants indicate whether the symptom was present for at least two weeks. IDD-L scores reflect the number of symptoms endorsed at a significant level for two weeks or more. 11% of participants surpassed the IDD-L screening threshold for a lifetime major depressive episode.

#### Social Interaction Anxiety Scale (SIAS)

The SIAS is a 20-item measure of tendencies toward social anxiety [72]. Items are rated on a scale ranging from 0 = “not at all characteristic of me” to 4 = “extremely all characteristic of me.” SIAS scores are the sum of items.

#### Social Anxiety Scale (SAS)

The SAS assesses fear and avoidance of situations that evoke social anxiety [73]. Participants are presented with a list of 24 situations (e.g., “speaking up at a meeting”). For each situation, they rate the degree to which they would feel fearful or anxious on a scale of 0 = “None” to 3 = “Severe” and how frequently they would avoid that situation on a scale of 0 = “Never” to 3 = “Always.” Total scores are the sum of the fear and avoidance ratings. 12% of participants were classified as moderately socially anxious and an additional 11% as markedly socially anxiety based on scores above 54 and 64, respectively [74].

#### Psychopathic Personality Inventory—Short Form (PPI-SF)

The PPI-SF is a 56-item self-report measure designed to assess psychopathic tendencies [75]. Items are rated on a scale ranging from 1 = “false” to 4 = “true.” Fearless Dominance scores are the sum of items (24 reverse-keyed) on the Fearlessness, Social Potency, and Stress Immunity subscales. We focus on the Fearless Dominance subscale here and provide analyses about the other factor (Self-Centered Impulsivity) and Coldheartedness in the S1 File.

#### Positive and Negative Activation Schedule (PANAS)

Participants completed items drawn from the factor-analytically supported PANAS scale [76, 77]: six positively valenced adjectives (i.e., determined, excited, enthusiastic, happy, proud, strong) and 10 negatively valenced adjectives (i.e., ashamed, depressed, distressed, guilty, hostile, irritable, nervous, sad, scared, upset). Participants rated the extent to which they were currently experiencing these emotions on a scale from 1 (slightly or not at all) to 5 (very much) before and after the dyadic interaction. Positive affect and negative affect scores were the average of corresponding items.

#### Physiological assessment

Physiological measurements were assessed continuously during a five-minute baseline period in which the participant sat quietly, during the dyadic interaction which lasted at least 5 minutes (Mn = 8.82 minutes, *SD* = 2.39 minutes), and during a five-minute post-dyad recovery period while the participant completed questionnaires. Sympathetic activity was indexed by skin conductance level (SCL) and parasympathetic activity was indexed by respiratory sinus arrhythmia (RSA).

SCL was acquired using 8-channel chassis BioLab acquisition software version 3.2 (Mindware Technologies LTD, Westerville, OH) at 10000 Hz through two disposable snap electrodes attached to the participant’s non-dominant palm. SCL was calculated using the Mindware EDA 2.10 Module (Mindware Technologies LTD, Westerville, OH). SCL values in each 30-second epoch below the expected range of values (<1 microSeimen), typically due to poor electrode placement or dry hands, were excluded.

RSA was acquired via electrocardiogram (ECG) with three disposable snap electrodes using a modified Lead II placement (right collar bone, left lower ribs, and right lower rib as ground). The signal was processed in Mindware HRV 2.10 Module (Mindware Technologies LTD, Westerville, OH) in 60-second epochs. The inter-beat interval (IBI) series was converted within the module to a time series with interpolation, resampled at a frequency of 10 samples per beat for the mean IBI interval per epoch. The data was then tapered with a Hamming window and a fast Fourier transform (FFT) was applied to derive a spectral distribution. RSA was quantified in milliseconds squared (3–10 ms^2^) by using the natural log of the integral power of the respiratory frequency band (0.12–0.40 Hz band). As has been recommended because of little sympathetic contribution to power, we used 0.12 Hz for the low frequency cutoff [78].

### Procedures

Procedures were approved by the university IRB before data collection began. After completing informed consent and baseline questionnaires covering psychopathology, participants were invited to complete laboratory procedures. A gender-matched member of the research team acted as a confederate “participant” for dyadic procedures. The confederates completed extensive training to standardize nonverbal and verbal cues for dominant and subordinate roles. The confederate and participant were greeted and completed written informed consent procedures upon arrival. After receiving instructions, confederates were taken to another room allegedly to complete parallel procedures.

Participants first completed the PANAS. To engage participants, the next task was (falsely) described as a measure of ability to accurately make personality judgments; participants were told that high scores on this task are associated with positive outcomes, including being well-liked by peers and having more satisfying relationships. For this task, participants were shown eight images of peoples’ eyes (from the revised Reading the Mind in the Eyes Test [79]) and asked to rate their gut intuition about the extent to which the person in the image was extraverted and conscientious on two 10-point scales. Participants were given 8 minutes to complete this task. Then, physiological sensors were attached to the participant.

Confederate’s ratings were completed based on participant ratings so that major discrepancies (at least 3 points different) appeared for four scores. For standardization, we created discrepancies for the same trait and image across all participants.

After baseline psychophysiological recording, the experimenter returned with the confederate, who also had sensors attached. (Although those sensors appeared to be gathering data, we could only record physiological data from the participant.) The experimenter then addressed the dyad together. In each dyad, one person was assigned the leader role and one the subordinate role (see S1 File for full script). The experimenter explained the role assigned to the participant first, and then the role assigned to the confederate. The “leader” was asked to sit in a large office chair on one side of a table and the “subordinate” was asked to sit in a small metal chair on the opposite side of the table. Dyads were told that the assignments were based on their questionnaire responses; in fact, assignments were random.

The experimenter then explained that there were discrepancies in ratings for quite a few pictures and asked the dyad to focus on the 4 largest discrepancies. Both members of the dyad were given their original rating sheets with the 4 most discrepant ratings circled. Participants were asked to discuss the discrepancies, beginning with their rationale for the original ratings, then deciding who made the more accurate rating, and recording a final consensus rating. Participants assigned to leader role were told they *got* to share their ratings and explanations first; participants assigned to subordinate roles were told they *had* to share their ratings and explanations first. Confederates were moved to another room after the interaction, and the participant completed post-interaction questionnaires while recovery physiological signals were recorded.

After the interaction, participants completed post-task PANAS ratings and rated their comfort level with their assigned role and relationship satisfaction (liked the partner, enjoyed working with the partner, you and partner worked well together). Ratings were made on a scale from 1 = “very slightly or not at all” to 5 = “extremely”.

At the end of the session, participants were asked if they felt deceived at any point of the study (2% endorsed) and if they believed the role assignments were made accurately (80% endorsed). Then, participants were debriefed regarding the hypotheses, the randomness of role assignments and the confederate.

### Data analysis

To reduce error variance, we created psychopathology composites based on scaled scores: mania as the mean of the HPS and the 7-Up (*r* = .60); depression as the mean of the IDD and the 7-Down (*r* = .65); social anxiety as the mean of the SIAS and the SAS (*r* = .67); and Fearless Dominance as the sum of 3 7-item PPI subscales of Social Potency, Stress Immunity, and Fearlessness [80], alpha = .79. As shown in Table 1, most psychopathology scores were low, in ranges expected for student samples. Distributions of variables were normal with the exceptions of positive affect ratings pre-task and negative affect ratings post-task.

**Table 1:** Descriptive statistics of key variables (N = 81).

	*Mean (SD)*	Scale Range	Skew	Kurtosis	α
*Psychopathology variables*					
HPS total	14.35 (4.30)	0–48	-0.11	-.43	.91
7U7D Mania subscale	10.97 (3.70)	7–28	1.16	0.99	.89
7U7D Depression subscale	11.51 (4.13)	7–28	0.98	1.44	.94
IDD-L	1.88 (2.25)	0–9	1.19	0.47	.86
SIAS	25.37 (12.20)	0–80	0.31	-0.37	.93
SAS	36.39 (20.44)	0–144	0.30	0.28	.88
PPI-SF Fearless dominance	17.52 (5.02)	7–28	0.07	-1.02	.79
*Dominance-related variables*					
PANAS–Positive (pre-task)	2.24 (0.66)	1–5	2.01	4.72	.88
PANAS–Negative (pre-task)	1.31 (0.48)	1–5	0.65	0.34	.84
PANAS–Positive (post-task)	2.18 (0.70)	1–5	0.65	0.27	.83
PANAS–Negative (post-task)	1.10 (0.16)	1–5	2.12	4.35	.83
Comfort in Assigned Role	3.18 (0.96)	1–5	-0.75	0.11	n/a
*Potential Confounds*					
Task Performance	1.44 (0.39)	1–2	0.24	-1.33	n/a
Relationship Quality–participant	3.35 (0.76)	1–5	-0.67	0.38	.71
Relationship Quality–confederate	3.41 (0.64)	1–5	-0.21	0.23	.83

Note. 7U7D = 7 Up 7 Down; HPS = Hypomanic Personality Scale; IDD = Inventory to Diagnose Depression; PANAS = Positive and Negative Affect Schedule; PPI-SF = Psychopathic Personality Inventory-Short Form; SAS = Social Anxiety Scale; SIAS = Social Interaction Anxiety Scale. Data were missing for 3–9 participants per scale.

As preliminary analyses, we considered zero-order correlations among these symptom composites. To evaluate potential confounds, we tested correlations of these composites and assigned role with gender, relationship quality and performance on the RMET.

We extracted key features of inter-individual variance in psychophysiological responding using functional principal components analysis fPCA; [81]). fPCA was applied separately to SCL and RSA signals. This involved re-representing the epoch-to-epoch physiology data as a set of continuous functions that capture central modes of variation over time, and then extracting principal component scores to use in analyses.

To assess hypotheses, we constructed multiple regression models with the four symptom composite variables, assigned role (0 = Subordinate, 1 = Leader), and interactions of symptom composites x role as independent variables. We computed parallel models for each dependent variable: positive affect, negative affect, role comfort, and each psychophysiology principal component score.

Analyses were conducted in R version 3.5.1 (R Foundation for Statistical Computing, Vienna, Austria). fPCA was implemented using the fdapace package [82]. Bootstrapped 95% confidence intervals were obtained using the boot package [83]. De-identified data is available at https://osf.io/2dufj/?view_only=2c80e29df50c425b9aab915e8335cffd.

## Results

### Preliminary analyses

Visual inspection and correlational analyses indicated that participants who endorsed suspected deception on debrief questions did not differ in positive or negative affect (controlling for baseline affect and role) or role comfort (controlling for role).

Psychopathology indices showed expected inter-correlations. Depression tendencies were significantly positively correlated with mania risk and social anxiety, *r*s = .26 and .30, respectively, and significantly negatively correlated with Fearless Dominance, *r* = -.36. Fearless Dominance scores also were significantly negatively correlated with social anxiety symptoms, *r* = -.62. Other psychopathology composites were not significantly correlated, all *r*s < |.20|. Female participants reported significantly higher social anxiety (*t*(72) = -2.13, *p* = .04) and lower Fearless Dominance scores (*t*(72) = 2.72, *p* = .008) than male participants, but did not differ on mania risk or depression, *t*(72) = .52, *p* = .61, and *t*(72) = -.36, *p* = .72, respectively.

Psychopathology tendencies were not confounded with baseline (pre-dyadic task) physiological activity, task performance, or participant or confederate ratings of relationship quality, all *r*s < |.20|. Random assignment to the experimental condition appeared effective in that participants assigned to the subordinate versus leader condition did not differ significantly on psychopathology composite scores (*t*s < |1.8|, *p*s > .08), relationship quality or task performance (*t*s < 1.7, *p*s > .10), or gender (χ^2^(1, 81) = .40, *p* = .64).

### Physiological activity components analysis

**Fig 1:**
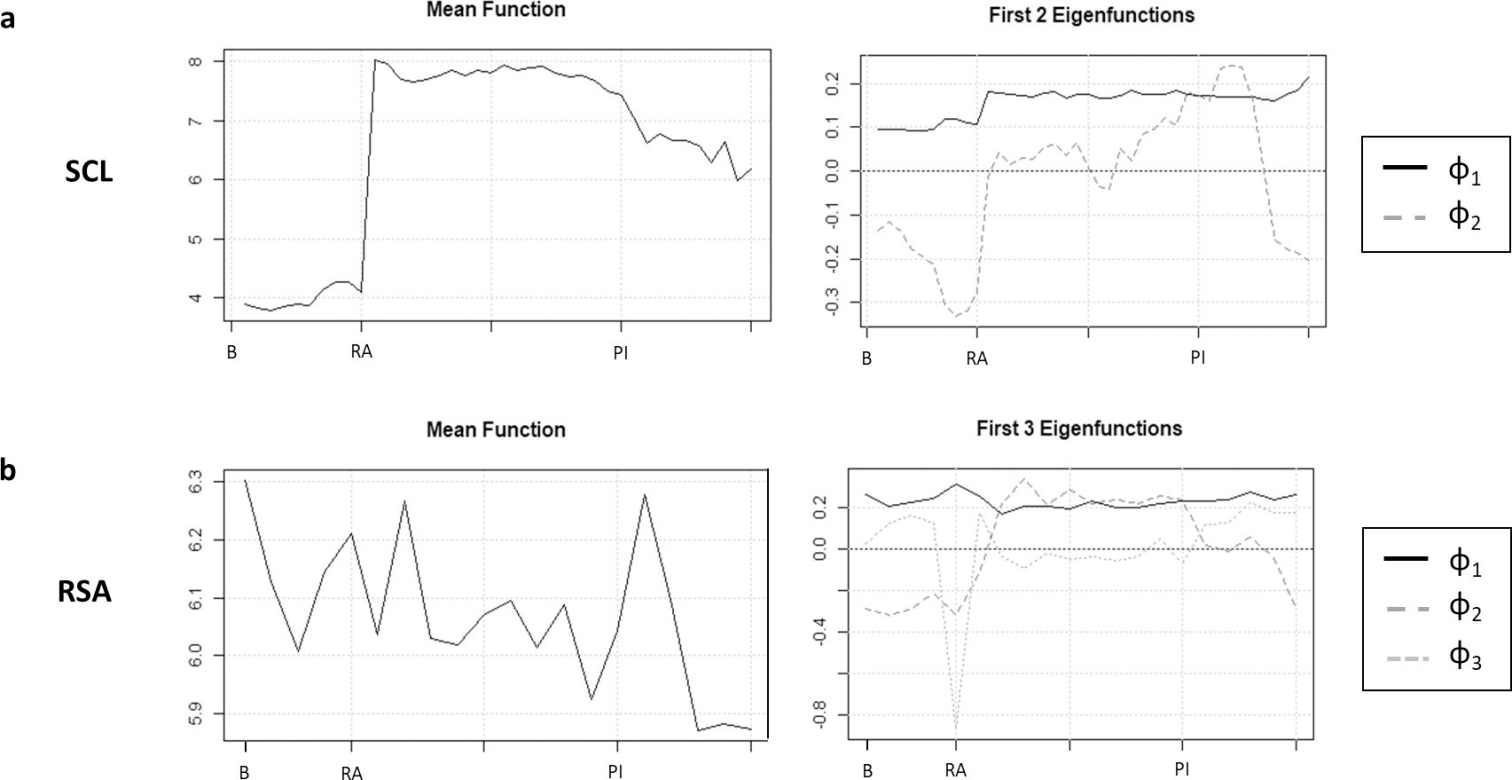
Mean functions and eigenfunctions for skin conductance and respiratory sinus arrythmia. The x-axis is time (in minutes). B = baseline; RA = roles assigned; PI = post-interaction.

Mean functions and corresponding eigenfunctions (reflecting change over time in relation to the mean function) from fPCA analyses are displayed in Fig 1.

For SCL, two components, which accounted for 95.7% of the variance in SCL, were retained. As shown in Fig 1A, fPCA1_SCL_ was an approximately constant function corresponding to the extent to which SCL was elevated from baseline to introduction of the role assignment and sustained throughout the recording period. fPCA2_SCL_ was an approximately quadratic function corresponding to increasing amplitude with time in the assigned role, and recovery after the interaction. In other words, fPCA1_SCL_ appeared to index tonic elevation, whereas fPCA2_SCL_ more closely indexed reactivity and recovery.

For RSA, three functional principal components were retained, which cumulatively accounted for 92.6% of the variance in RSA. As shown in Fig 1B, fPCA1_RSA_ was an approximately constant function corresponded to higher RSA throughout the recording period relative to the mean, comparable to fPCA1_SCL_. fPCA2_RSA_ was an approximately quadratic function corresponded to the degree to which RSA rose or fell with the introduction of the role assignment and was sustained at that level throughout the dyadic task. Conversely, fPCA3_RSA_ corresponded to the degree of temporary diminishment in RSA at the time of role assignment relative to baseline. In other words, fPCA2_RSA_ and fPCA3_RSA_ appeared to reflect two separable components of reactivity to the experimental manipulation, with fPCA2_RSA_ reflecting a sustained response throughout the dyadic interaction whereas fPCA3_RSA_ reflecting an acute response to the role assignment. For ease of interpretability, fPCA3_RSA_ was reversed so that higher scores reflected higher RSA.

In summary, fPCA1_SCL_ and fPCA1_RSA_ index more stable differences in the amplitude of physiological activity across these two channels, whereas fPCA2_SCl_, fPCA2_RSA,_ and fPCA3_RSA_ more closely track reactivity. Because our focus was on psychophysiological response to the experimental induction, we focus on fPCA2_SCL,_ fPCA2_RSA_, and fPCA3_RSA_ here. More detailed graphs of each eigenfunction, along with analyses of the two psychophysiological factors reflecting tonic elevations—fPCA1_SCL and_ fPCA1_RSA_—are provided in the S1 File.

Dependent variables were largely independent. The only statistically significant correlations were the two psychophysiological indices of reactivity throughout the experimental induction—fPCA2_SCL_ with fPCA2_RSA_, *r* = -.26, and post-task positive affect with role comfort, *r* = .24, *p*s < .05. All other correlations among dependent variables were < |.20|.

### Affect and role comfort

We computed three regression models to evaluate main and interactive effects of the four psychopathology tendencies and assigned role on positive affect, negative affect, and comfort with assigned role (Given the non-normal distributions of affect, Box-Cox transformations were applied to these variables and models were recomputed. The results were interpretively identical.). As shown in Table 2, significant main effects of social anxiety and Fearless Dominance scores were observed for post-task negative affect, controlling for baseline negative affect, such that participants with higher social anxiety and Fearless Dominance scores tended to report higher post-task negative affect regardless of assigned condition. For post-task positive affect, significant main effects of depression and Fearless Dominance were observed, such that higher scores on those measures were associated with lower post-task positive affect.

**Table 2:** Multiple regression models of role, psychopathology tendencies, and their interactions on negative affect, positive affect, role comfort and psychophysiological variables.

		*β*	95% CI	*p*
Negative Affect				
	Pre-task Negative Affect	.08	-.16, .33	.58
	Assigned Role	-.07	-.47, .42	.62
	Mania Composite	.09	-.25, .39	.66
	Fearless Dominance	**.60**	**.09, 1.21**	**.03**
	Depression Composite	.14	-.38, .66	.52
	Social Anxiety Composite	**.68**	**.08, 1.14**	**.004**
	Role x Mania	.01	-.47, .53	.95
	Role x Fearless Dominance	-.34	-1.13, .49	.16
	Role x Depression	.12	-.57, .93	.58
	Role x Social Anxiety	-.30	-1.15, .58	.16
Positive Affect				
	Pre-task Positive Affect	-.14	-.41, .09	.26
	Assigned Role	.09	-.28, .84	.52
	Mania Composite	.15	-.18, .46	.4
	Fearless Dominance	**-.60**	**-.93, -.26**	**.02**
	Depression Composite	**-.46**	**-.88, -.17**	**.03**
	Social Anxiety Composite	-.04	-.40, .27	.86
	Role x Mania	**-.49**	**-1.15, -.16**	**.01**
	Role x Fearless Dominance	.26	-.09, 1.09	.09
	Role x Depression	**.49**	**.19, 1.22**	**.02**
	Role x Social Anxiety	.01	-.53, .58	.94
Role Comfort				
	Assigned Role	.04	-.61, .57	.77
	Mania Composite	**.42**	**.07, .89**	**.03**
	Fearless Dominance	.13	-.40, .61	.64
	Depression Composite	-.37	-.88, -.03	.08
	Social Anxiety Composite	**-.49**	**-.90, -.06**	**.03**
	Role x Mania	**-.41**	**-1.15, -.04**	**.03**
	Role x Fearless Dominance	-.15	-.85, .43	.53
	Role x Depression	.41	-.00, 1.07	.05
	Role x Social Anxiety	-.13	-.95, .47	.52
SCL fPCA2				
	Assigned Role	-.18	-.65, .52	.27
	Mania Composite	-.08	-.53, .45	.73
	Fearless Dominance	.17	-.52, .65	.57
	Depression Composite	-.09	-.68, .36	.69
	Social Anxiety Composite	.29	-.51, .81	.27
	Role x Mania	.08	-.61, .65	.73
	Role x Fearless Dominance	.06	-.63, .85	.83
	Role x Depression	.02	-.61, .65	.92
	Role x Social Anxiety	-.38	-1.19, .20	.12
RSA fPCA2				
	Assigned Role	.03	-.55, .59	.84
	Mania Composite	.08	-.36, .78	.73
	Fearless Dominance	-.02	-.61, .44	.95
	Depression Composite	.02	-.41, .47	.93
	Social Anxiety Composite	-.06	-.60, .57	.81
	Role x Mania	.10	-.60, .69	.65
	Role x Fearless Dominance	-.03	-.83, .63	.41
	Role x Depression	.31	-.26, 1.03	.23
	Role x Social Anxiety	-.28	-1.27, .40	.24
RSA fPCA3				
	Assigned Role	.11	-.25, .62	.46
	Mania Composite	**-.48**	**-.64, -.01**	**.05**
	Fearless Dominance	.22	-.36, .68	.46
	Depression Composite	.03	-.38, .64	.92
	Social Anxiety Composite	-.12	-.53, .33	.48
	Role x Mania	**.45**	**.01, .80**	**.05**
	Role x Fearless Dominance	-.27	-.80, .44	.31
	Role x Depression	-.06	-.65, .46	.82
	Role x Social Anxiety	-.29	-.87, .27	.23

Note. 95% confidence intervals were bootstrapped to increase robustness against violations of multivariate distributional assumptions in the context of small samples.

**Fig 2:**
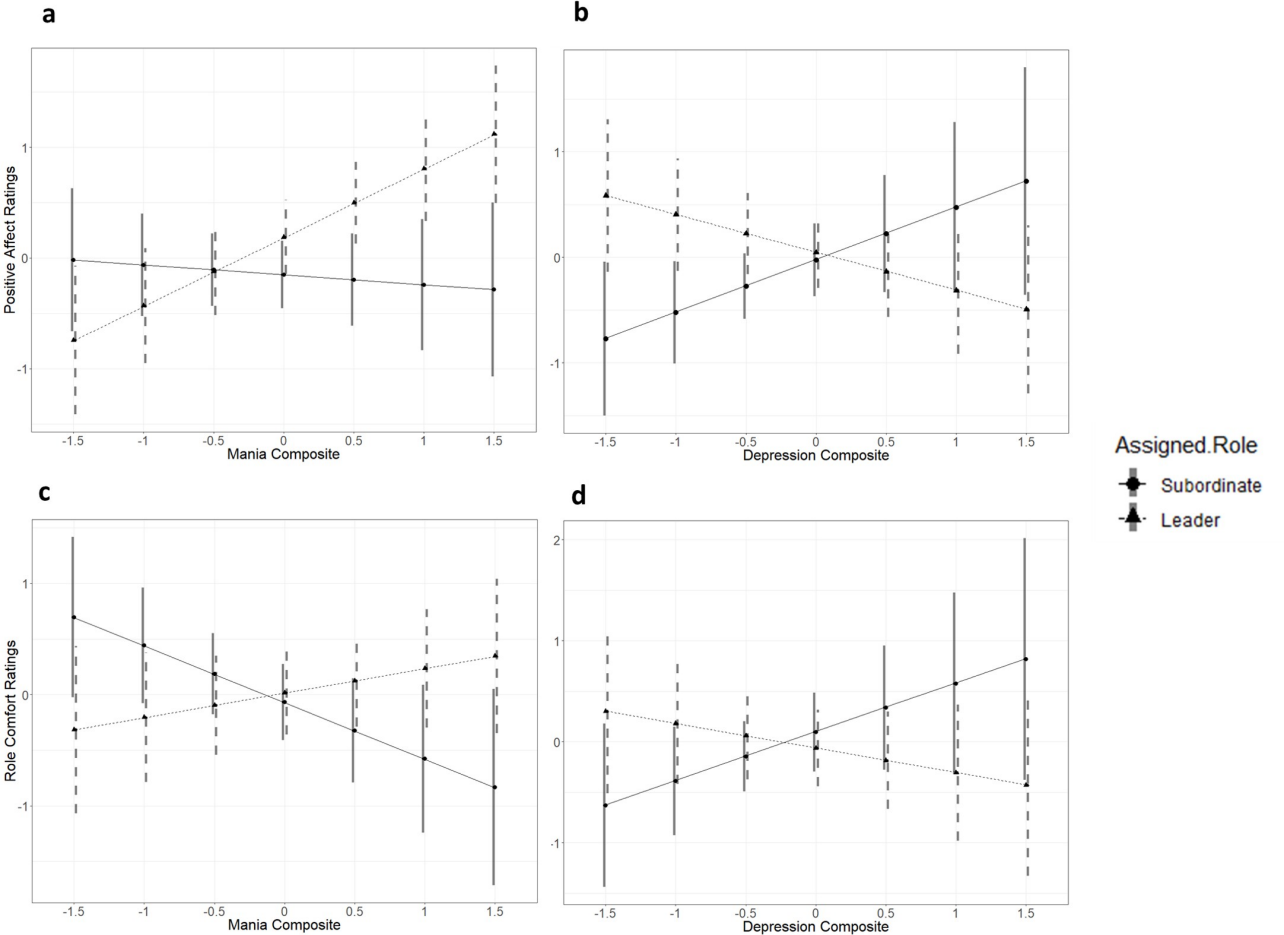
Interactions of assigned role with mania risk and depressive symptoms predict post-task positive affect, controlling for pre-task positive affect, and self-reported role comfort.

Beyond these main effects, for positive affect, significant interaction effects were observed for assigned role with mania risk and with depression symptoms. As shown in Fig 2A, higher mania risk predicted higher positive affect for participants assigned to the leader role (*b* = .51, 95% CI = [.22, .81]), whereas no significant relationship was observed between mania risk and positive affect for participants assigned to the subordinate role (*b* = -.15, 95% CI = [-.51, .21]). Conversely, as shown in Fig 2B, higher depression severity predicted higher positive affect for participants assigned to the subordinate condition (*b* = .37, 95% CI = [.05, .80], whereas no significant effect was observed for participants assigned to the leader condition (*b* = -.18, 95% CI = [-.51, .15]).

With respect to comfort with the assigned role, main effects of social anxiety symptoms and mania risk were observed. That is, higher social anxiety predicted less role comfort regardless of role assignment, whereas higher mania risk predicted more comfort. We also observed significant interactions of assigned role with mania risk and depression scores. Higher mania risk predicted significantly *less* comfort for participants assigned to the subordinate role (*b* = -.46, 95% CI = [-.87, -.05]), but not for participants assigned to the leader role (*b* = .15, 95% CI = [-.20, .50]), as shown in Fig 2C. Conversely, as shown in Fig 2D, higher depression severity predicted marginally greater role comfort for participants assigned to the subordinate role (*b* = .42, 95% CI = [-.06, .91]), but marginally lower comfort for those assigned to the leader role (*b* = -.17, 95% CI = [-.56, .22]), although neither of these simple slopes was statistically significant.

### Psychophysiology fPCA patterns

Paralleling analyses of affect and role comfort, we conducted 3 multiple regression models with the functional PCA scores capturing reactivity to experimental induction as DVs. No significant effects were observed for fPCA2_SCL_.

**Fig 3:**
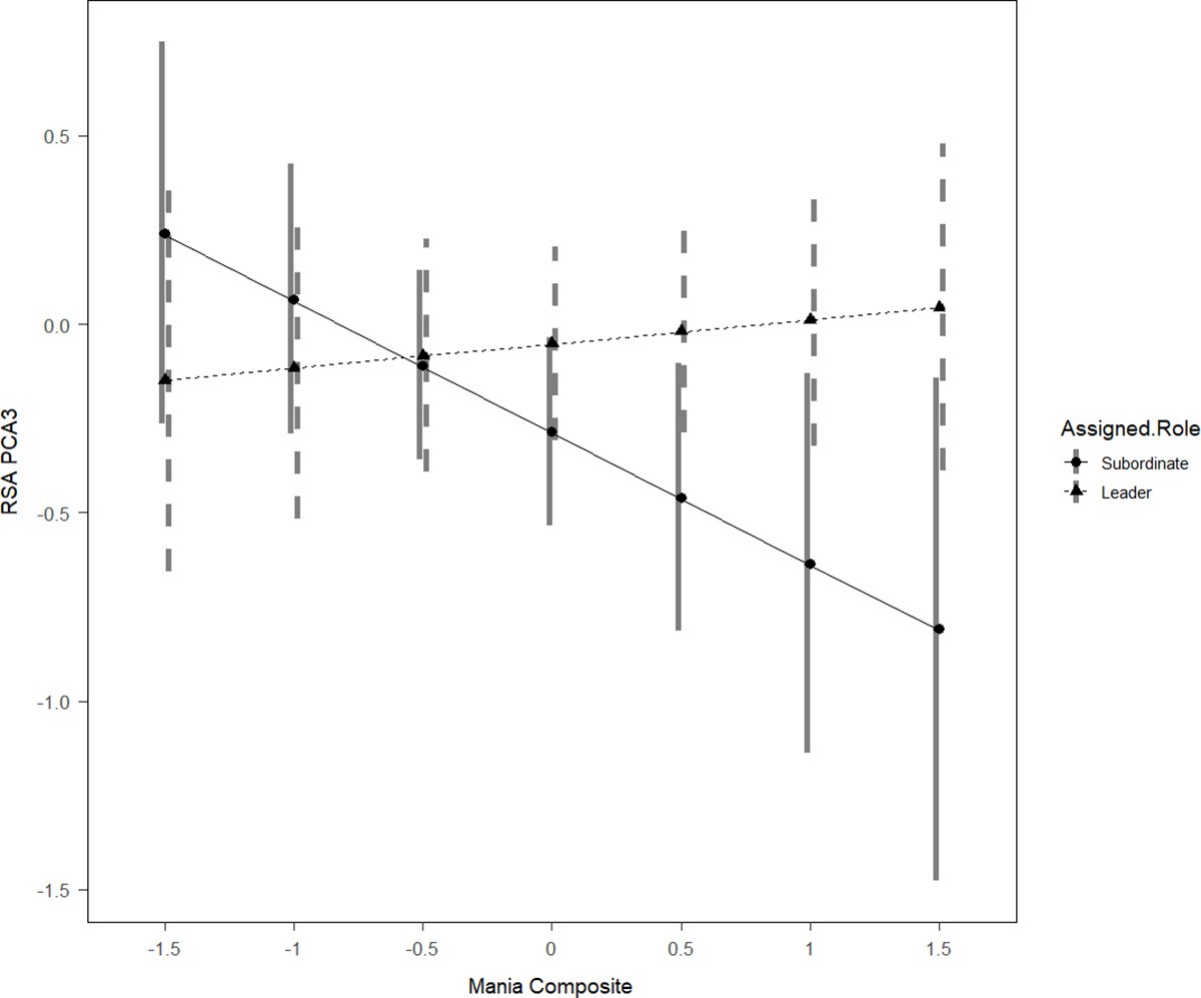
Interaction between assigned role and mania risk predicts respiratory sinus arrythmia.

With respect to the RSA response to role assignment, as indexed by fPCA3_RSA)_, significant main effects of mania risk and Fearless Dominance were observed such that higher mania risk scores predicted a greater decrement in RSA whereas Fearless Dominance predicted increased RSA immediately after role assignment regardless of role. The mania risk effect was qualified by a significant interaction of manic tendencies with role, as shown in Fig 3. Decomposition of the simple slopes indicated that this main effect was driven primarily by participants assigned to the subordinate role (*b* = -.23, 95% CI = [-.47, .01] and not participants assigned to the leader role (*b* = .02, 95% CI = [-.15, .21]), although neither of these slopes was statistically significant. No significant effects were observed for fPCA2_RSA_.

#### Post-hoc analyses

At the request of reviewers, we provide more detail regarding interactions with gender, as well as more refined analyses of the PPI and HPS subscales, in the S1 File. As shown in S1 Table in S1 File, gender did not show significant three-way interactions with any of the psychopathology indices x role assignment effects, although power was limited to examine 3-way interactions. Men appeared to show diminished RSA immediately after assignment to the subordinate role as compared to the leader role, whereas women appeared to show diminished RSA immediately after assignment to the leader role, perhaps suggesting differential comfort for men and women to these roles. Other effects appeared to be substantially consistent for men and women.

Regarding subscale analyses, there was no evidence that psychopathy (PPI) subscales other than Fearless Dominance were related to reactivity to experimental condition, as shown in S2 Table in S1 File. The three subscales of the HPS, on the other hand, were differentially associated with key outcomes, such that Social Vitality predicted participants’ self-reported negative affect and role discomfort in the subordinate role compared to the dominant role, whereas Mood Volatility and Excitement were linked to physiological indices of reactivity.

## Discussion

The current study provides several novel contributions to the field, as the first test of how multiple forms of psychopathology guide multimodal responses to a well-validated experimental manipulation of dominance roles. Effects did not appear to be confounded by relationship with the confederate, nor by problems enacting the task.

Consistent with hypotheses, manic and depressive tendencies related to responses to dominance roles across channels. Manic tendencies related to more decline in positive affect, more discomfort, and a larger RSA decline after assignment to the subordinate role as compared to the leader role. Conversely, higher depression scores related modestly to a more positive response to the subordinate role than the leadership role, including more positive affect and more role comfort. Taken together, findings indicate that manic symptoms relate to discomfort with subordinate roles compared to leadership roles, whereas depressive symptoms relate to discomfort with leadership compared to subordinate roles. Although consistent effects for both mania risk and depression emerged across separate outcomes, replication will be important given the potential for type I error with the five regression models conducted.

In contrast with manic and depressive syndromes, we observed no significant effects of role assignment related to Fearless Dominance or social anxiety. Contrasting with previous findings of sensitivity to subordination, socially anxious individuals reported more discomfort regardless of the experimentally assigned role. This generalized discomfort with the social interaction is consistent with the definition of the disorder and with some previous findings [56]. Participants’ awareness of the social nature of the tasks before the baseline psychophysiological recording began may have blunted post-baseline reactivity. For those with higher social anxiety, the salience of interacting with a stranger likely over-rode the experimental manipulation.

Fearless Dominance scores were related to higher negative and lower positive affect but no differential reactivity to experimental condition. S1 File showed similar null effects for the other psychopathy (PPI) subscales. PPI subscales showed only modest reliability, which could relate to the null findings. Previous work has examined the expression of dominance in real-world settings, including aggressive responses to disrespect [36], which might be a more sensitive approach. Psychopathy has also been tied to substantial blunting of psychophysiological reactivity to interpersonal stimuli [84], and so the use of psychophysiological indices may have been problematic. Theory also suggests that Fearless Dominance may lead to poor outcomes only when disinhibited and antisocial features of psychopathy are present [85], and we were underpowered to examine these interactions.

Other limitations are important across psychopathologies. With our small sample size, the current study had 80% power to observe moderate effect sizes (f^2^ > = .10), but more limited to detect 3-way interactions, such as those with gender [86]. Given this small sample size, there is a clear need to conduct further research to assess replicability of findings. Although we attempted to oversample students with high scores on psychopathology indices, our college student sample still showed relatively modest symptom levels, which may have contributed to the null effects for Fearless Dominance; that we observed pronounced effects of depressive and manic tendencies even at mild levels is intriguing. The small range of affect ratings could limit statistical power to examine affective response. It also would be wise to consider testosterone or testosterone-cortisol responses to dominance challenges [87, 88] rather than the psychophysiological indices we relied on here, particularly as we saw no evidence that skin conductance was sensitive to the experimental induction. Although our study was not longitudinal, our findings fit with previous longitudinal and experimental demonstrations of dominance profiles in depression and mania [89, 90].

Notwithstanding the limitations, the current study was novel in considering multiple dimensions of psychopathology using multi-modal measures of reactivity to a well-validated experimental manipulation of dominance and subordination. Findings indicated that manic tendencies related to discomfort with subordination, whereas depressive symptoms related to more comfort with subordination than leadership. Current findings suggest the importance of the dominance system in the mood disorders, and the merit of using laboratory procedures to consider these profiles.

## Supporting information

S1 Fig (PNG)

S2 Fig (PNG)

S3 Fig (TIF)

S1 File (DOCX)
