# Supplementary figures and images for: Social dominance and multiple dimensions of psychopathology: An experimental test of reactivity to leadership and subordinate roles
Source: PLoS One. 2021 Apr 28;16(4):e0250099. doi: 10.1371/journal.pone.0250099 (PMC8081185; doi:10.1371/journal.pone.0250099)

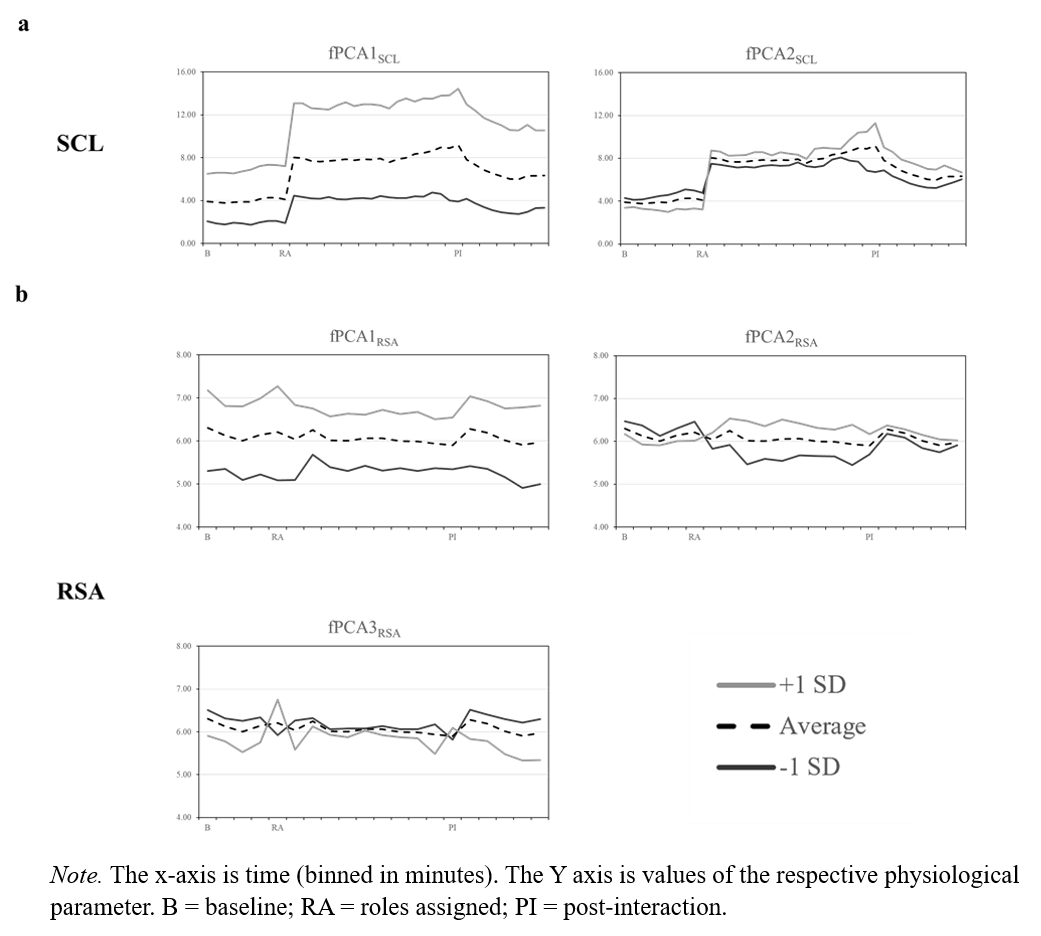

Supplement: S1 Fig — (PNG) [file pone.0250099.s001.png]

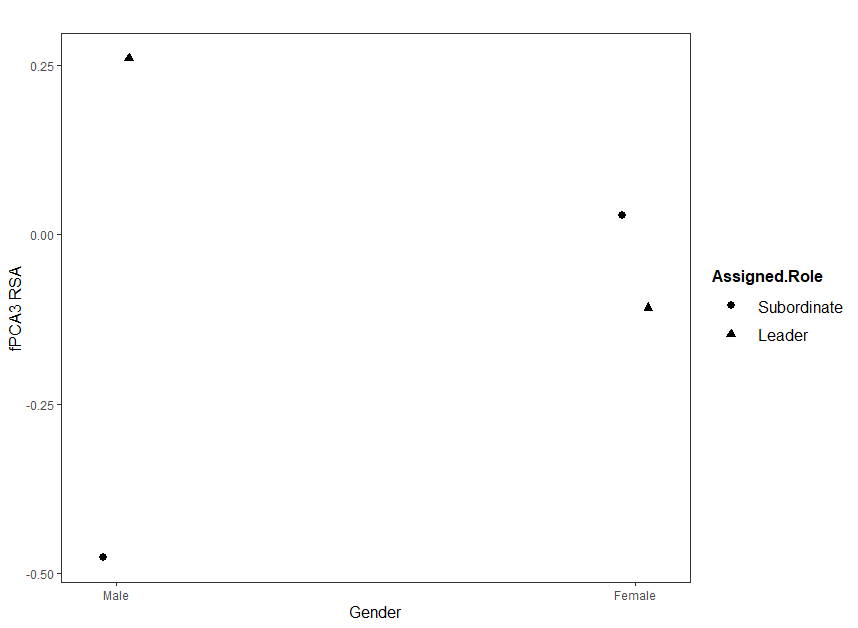

Supplement: S2 Fig — (PNG) [file pone.0250099.s002.png]

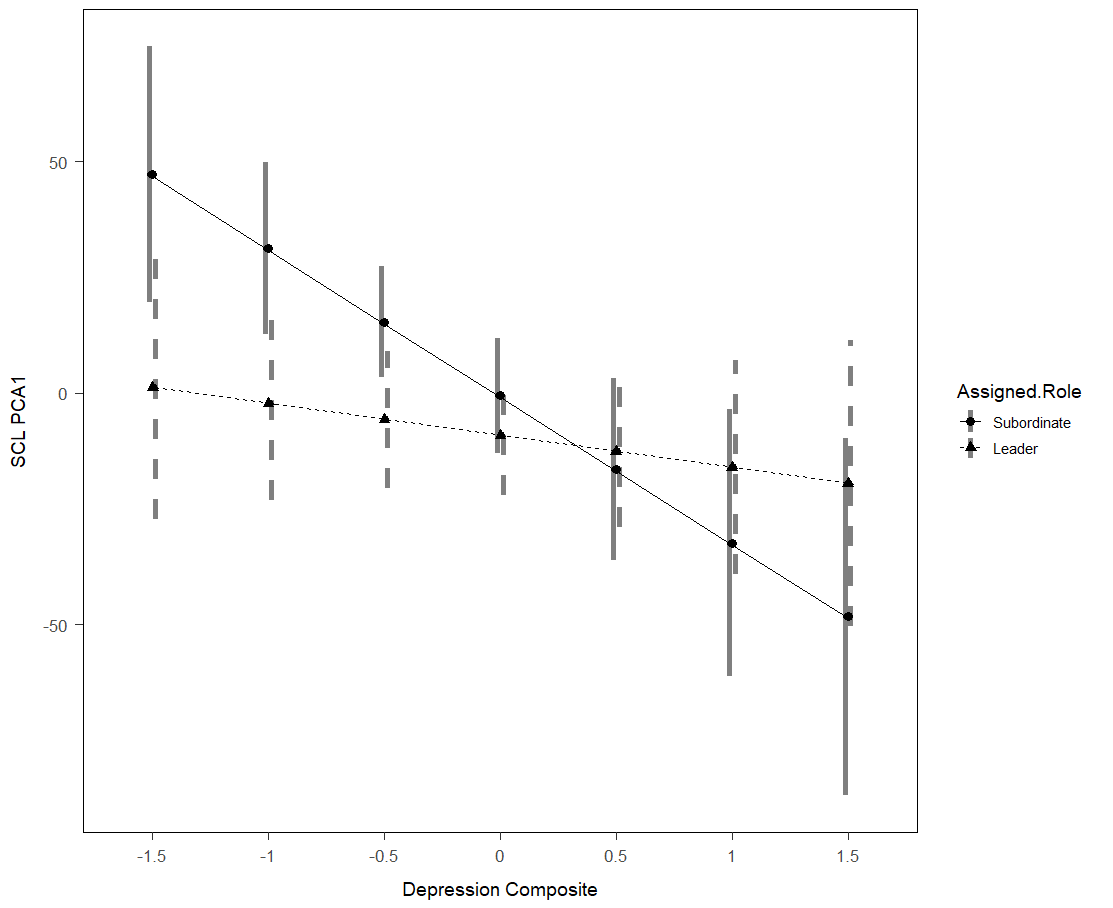

Supplement: S3 Fig — (TIF) [file pone.0250099.s003.tif]
