# Supplementary material for: Social dominance and multiple dimensions of psychopathology: An experimental test of reactivity to leadership and subordinate roles
Source: PLoS One. 2021 Apr 28;16(4):e0250099. doi: 10.1371/journal.pone.0250099 (PMC8081185; doi:10.1371/journal.pone.0250099)
Supplement: S1 File — (DOCX) [file pone.0250099.s004.docx]

**S1 File**

The goal of this supplement is to provide more detailed graphs regarding our psychophysiology PCA factor scores, to provide analyses of the two psychophysiological factors reflecting tonic elevations_,_ to provide more details on post-hoc analyses that were statistically underpowered, and to provide the script for the dyadic task.

Figure S1 provides the functional PCA eigenvalues graphed by minutes in the study.

**Figure S1**

*Functional PCA eigenvalues graphed by minutes in the study.*

**Supplementary Analyses**

At the request of reviewers, several supplementary, post-hoc analyses were conducted and included here both for the sake of completeness and as exploratory signposts for future research. More specifically, first, we examined the three-way interactions of role by psychopathology by gender. Second, we expanded our analysis of psychopathy beyond Fearless Dominance to include Self-Centered Impulsivity and Coldheartedness. Third, we conducted analyses of three factor-analytically derived subscales of the HPS in a bid toward greater specificity with respect to mania findings.

Each of the analyses is parallel to those provided in the main body of the paper, in that we conducted multiple regression models with psychopathology indices, assigned role (0 = Subordinate, 1 = Leader), and interactions of symptom composites x role as independent variables. Analyses of gender were identical to those reported in the main manuscript, but with additional regressors added to for the interactions between gender, role, and psychopathology indices. Analyses of the psychopathy subscales examined the conjoint main effects of the three subscales, and the interactive effects with assigned role. Similarly, analyses of the mania risk (HPS) subscales examined the conjoint main and interactive effects of the three subscales.

**Supplementary Method.** For supplementary analyses of psychopathy, the Self-Centered Impulsivity higher-order factor was computed as the sum of four 7-item subscales of the PPI-SF: Carefree Nonplanfulness, Impulsive Nonconformity, Machiavellian Egocentricity, and Blame Externalization (alpha = .77). The Coldheartedness subscale has previously been found not to load onto either Fearless Dominance or Self-Centered Impulsivity; instead, Coldheartedness consists of 7 items on the PPI-SF that index lack of sentimentality, empathic concern, and openness (alpha = .79) [1]. Fearless Dominance, Self-Centered Impulsivity, and Coldheartedness were significantly, but modestly inter-correlated, *r*s ranging from .24 to .30.

For supplementary analyses of mania risk, we examined three factor-analytically subscales of the HPS [2]. The Sociality Vitality subscale consists of 22 items related to sociability, assertiveness, and persuasiveness (alpha = .82). The Mood Volatility subscale consists of 15 items related to affective lability, excessive energy, irritability, and racing thoughts (alpha = .84). The Excitement subscale consists of 8 items related to high arousal positive affectivity (alpha = .82). The three HPS subscales were more substantially inter-correlated, *r*s ranging from .49 to .68.

**Supplementary Results.**

*Gender.* Given the sample size, it is perhaps unsurprising that few significant three-way role by psychopathology by gender interactions were observed, as shown in Table S1. As shown, we did observe a significant interaction of gender x assigned role in RSA fPCA3. Among men, fPCA3 was substantially lower, indicating lower RSA immediately after the role assignment was explained, for those assigned to the subordinate role than the leader role. In contrast, among women, fPCA3 indicated that RSA response immediately after the role assignment was modestly lower among those assigned to the leader role than the subordinate role. This interaction is shown graphically in Figure S2.

Table S1.

*Multiple regression models of role, psychopathology tendencies, gender, and their interactions on negative affect, positive affect, role comfort and psychophysiological reactivity variables.*

|  |  | *β* | 95% CI | *p* |
| --- | --- | --- | --- | --- |
| Negative Affect |  |  |  |  |
|  | Pre-Task Negative Affect | .02 | -0.27, 0.32 | .87 |
|  | Assigned Role | -.44 | -1.71, 0.83 | .49 |
|  | Gender | .19 | -0.59, 0.96 | .63 |
|  | Mania Composite | .13 | -0.56, 0.82 | .71 |
|  | Fearless Dominance | .35 | -1.20, 1.90 | .65 |
|  | Depression Composite | .27 | -.51, .106 | .48 |
|  | Social Anxiety Composite | .66 | -0.49, 1.81 | .25 |
|  | Role x Gender | .07 | -1.39, 1.52 | .93 |
|  | Role x Mania | .24 | -0.64, 1.12 | .59 |
|  | Role x Fearless Dominance | -.57 | -2.44, 1.29 | .54 |
|  | Role x Depression | .42 | -.90, 1.73 | .53 |
|  | Role x Social Anxiety | -.82 | -2.40, .77 | .31 |
|  | Gender x Mania | .04 | -0.81, 0.88 | .93 |
|  | Gender x Fearless Dominance | .18 | -1.49, 1.86 | .83 |
|  | Gender x Depression | .85 | -0.18, 1.88 | .93 |
|  | Gender x Social Anxiety | -.13 | -1.40, 1.14 | .84 |
|  | Role x Gender x Mania | -.26 | -1.39, 0.87 | .65 |
|  | Role x Gender x Fearless Dominance | .23 | -1.81, 2.27 | .82 |
|  | Role x Gender x Depression | .34 | -1.19, 1.86 | .66 |
|  | Role x Gender x Social Anxiety | .37 | -1.43, 2.16 | .68 |
| Positive Affect |  |  |  |  |
|  | Pre-Task Positive Affect | -.13 | -0.43, 0.16 | .36 |
|  | Assigned Role | .04 | -0.79, 1.54 | .52 |
|  | Gender | .42 | -0.27, 1.12 | .23 |
|  | Mania Composite | .29 | -.34, .91 | .36 |
|  | Fearless Dominance | -.52 | -2.16, 1.13 | .53 |
|  | Depression Composite | -.45 | -1.17, .27 | .21 |
|  | Social Anxiety Composite | -.24 | -1.38, .90 | .67 |
|  | Role x Gender | -.62 | -1.96, 0.73 | .36 |
|  | Role x Mania | **-.91** | **-1.69, .12** | **.03** |
|  | Role x Fearless Dominance | .48 | -1.40, 2.37 | .61 |
|  | Role x Depression | .92 | -.26, 2.10 | .13 |
|  | Role x Social Anxiety | .72 | -.80, 2.23 | .35 |
|  | Gender x Mania | .23 | -0.54, 1.00 | .55 |
|  | Gender x Fearless Dominance | .05 | -1.69, 1.79 | .95 |
|  | Gender x Depression | -.62 | -0.87, 1.06 | .85 |
|  | Gender x Social Anxiety | -.44 | -1.69, 0.80 | .48 |
|  | Role x Gender x Mania | -.41 | -1.43, 0.61 | .43 |
|  | Role x Gender x Fearless Dominance | -.18 | -2.24, 1.88 | .86 |
|  | Role x Gender x Depression | .25 | -1.13, 1.64 | .71 |
|  | Role x Gender x Social Anxiety | 1.12 | -0.55, 2.79 | .18 |
| Role Comfort |  |  |  |  |
|  | Assigned Role | .66 | -0.59, 1.91 | .30 |
|  | Gender | -.41 | -1.17, 0.35 | .28 |
|  | Mania Composite | .16 | -.52, .85 | .64 |
|  | Fearless Dominance | .03 | -1.48, 1.55 | .96 |
|  | Depression Composite | -.30 | -1.07, .44 | .44 |
|  | Social Anxiety Composite | -.12 | -1.24, 1.01 | .84 |
|  | Role x Gender | -.59 | -2.03, 0.85 | .42 |
|  | Role x Mania | -.13 | -.99, .74 | .77 |
|  | Role x Fearless Dominance | -.50 | -2.32, 1.32 | .58 |
|  | Role x Depression | .10 | -1.20, 1.40 | .88 |
|  | Role x Social Anxiety | .49 | -1.09, 2.06 | .54 |
|  | Gender x Mania | -.40 | -1.24, 0.44 | .34 |
|  | Gender x Fearless Dominance | -.09 | -1.72, 1.54 | .91 |
|  | Gender x Depression | -.10 | -1.11, 0.91 | .85 |
|  | Gender x Social Anxiety | -.35 | -1.61, 0.90 | .57 |
|  | Role x Gender x Mania | .71 | -0.41, 1.83 | .21 |
|  | Role x Gender x Fearless Dominance | .83 | -1.15, 2.82 | .40 |
|  | Role x Gender x Depression | -.15 | -1.66, 1.37 | .85 |
|  | Role x Gender x Social Anxiety | -.47 | -2.24, 1.31 | .60 |
| SCL fPCA2 |  |  |  |  |
|  | Assigned Role | -.86 | -2.39, 0.67 | .26 |
|  | Gender | .05 | -0.90, 0.99 | .92 |
|  | Mania Composite | -.55 | -1.61, 0.50 | .30 |
|  | Fearless Dominance | .06 | -1.74, 1.86 | .94 |
|  | Depression Composite | -.12 | -1.03, .78 | .79 |
|  | Social Anxiety Composite | .17 | -.21, 1.54 | .81 |
|  | Role x Gender | .74 | -1.00, 2.48 | .40 |
|  | Role x Mania | .76 | -0.47, 1.99 | .22 |
|  | Role x Fearless Dominance | .43 | -1.74, 2.61 | .69 |
|  | Role x Depression | -.69 | -2.30, 0.93 | .40 |
|  | Role x Social Anxiety | -.82 | -2.82, 1.19 | .42 |
|  | Gender x Mania | .60 | -0.60, 1.79 | .32 |
|  | Gender x Fearless Dominance | .09 | -1.84, 2.03 | .92 |
|  | Gender x Depression | -.23 | -1.41, 0.96 | .70 |
|  | Gender x Social Anxiety | .60 | -0.96, 2.16 | .44 |
|  | Role x Gender x Mania | -.92 | -2.39, 0.55 | .21 |
|  | Role x Gender x Fearless Dominance | -.49 | -2.87, 1.88 | .68 |
|  | Role x Gender x Depression | .65 | -1.22, 2.51 | .49 |
|  | Role x Gender x Social Anxiety | .21 | -2.06, 2.49 | .85 |
| RSA fPCA2 |  |  |  |  |
|  | Assigned Role | .69 | -0.41, 1.78 | .21 |
|  | Gender | -.34 | -1.16, 0.49 | .42 |
|  | Mania Composite | -.03 | -1.05, 0.98 | .95 |
|  | Fearless Dominance | -.03 | -1.68, 1.63 | .97 |
|  | Depression Composite | -.52 | -1.39, 0.36 | .24 |
|  | Social Anxiety Composite | .22 | -1.06, 1.50 | .73 |
|  | Role x Gender | -.82 | -2.11, 0.47 | .21 |
|  | Role x Mania | -.12 | -1.32, 1.09 | .85 |
|  | Role x Fearless Dominance | -.08 | -1.98, 1.81 | .93 |
|  | Role x Depression | **1.26** | **0.17, 2.36** | **.03** |
|  | Role x Social Anxiety | -.24 | -1.91, 1.44 | .78 |
|  | Gender x Mania | .29 | -0.85, 1.42 | .61 |
|  | Gender x Fearless Dominance | -.06 | -1.82, 1.70 | .94 |
|  | Gender x Depression | .93 | -0.18, 2.03 | .10 |
|  | Gender x Social Anxiety | -.39 | -1.81, 1.02 | .58 |
|  | Role x Gender x Mania | -.23 | -1.65, 1.18 | .74 |
|  | Role x Gender x Fearless Dominance | -.13 | -2.19, 1.93 | .90 |
|  | Role x Gender x Depression | -1.37 | -2.75, 0.01 | .05 |
|  | Role x Gender x Social Anxiety | -.72 | -2.60, 1.15 | .44 |
| RSA fPCA3 |  |  |  |  |
|  | Assigned Role | **1.12** | **0.08, 2.17** | **.04** |
|  | Gender | .77 | -0.01, 1.55 | .05 |
|  | Mania Composite | -.01 | -.98, .95 | .98 |
|  | Fearless Dominance | 1.10 | -0.48, 2.68 | .17 |
|  | Depression Composite | -.09 | -0.92, 0.75 | .83 |
|  | Social Anxiety Composite | -.41 | -1.63, .81 | .50 |
|  | Role x Gender | **-1.33** | **-2.56, -0.10** | **.04** |
|  | Role x Mania | .05 | -1.09, 1.20 | .92 |
|  | Role x Fearless Dominance | -1.55 | -3.35, 0.26 | .09 |
|  | Role x Depression | .03 | -.101, 1.07 | .96 |
|  | Role x Social Anxiety | -.14 | -1.73, 1.45 | .86 |
|  | Gender x Mania | -.51 | -1.59, 0.57 | .35 |
|  | Gender x Fearless Dominance | -1.15 | -2.83, 0.53 | .17 |
|  | Gender x Depression | .92 | -0.13, 1.97 | .08 |
|  | Gender x Social Anxiety | -.54 | -1.89, 0.81 | .42 |
|  | Role x Gender x Mania | .65 | -0.70, 1.99 | .34 |
|  | Role x Gender x Fearless Dominance | 1.46 | -0.50, 3.42 | .14 |
|  | Role x Gender x Depression | -.43 | -1.74, 0.88 | .52 |
|  | Role x Gender x Social Anxiety | -.50 | -2.28, 1.29 | .58 |

*Note. 95% confidence intervals were bootstrapped to increase robustness against violations of multivariate distributional assumptions in the context of small samples.*

**Figure S2**

*Interaction between assigned role and gender predicts RSA (RSA PCA3) patterns*

*Psychopathy (PPI) subscales.* No significant interactions of assigned role with any of the three PPI-SF variables were observed, as reported in Table S2. A main effect of Fearless Dominance on post-task positive affect was observed, consistent with the results of primary study analyses.

*Mania Risk (HPS) Subscales.* Somewhat parallel with the primary study analyses indicating that mania risk scores interacted with assigned role to predict (positive) affect and comfort, analyses of HPS subscales indicated that the Social Vitality interacted with assigned role to predict (negative) affect and comfort, as shown in Table S3. That is, significant role by Social Vitality interactions were observed for two of the three self-report indices: post-task negative affect and role comfort. More specifically, for participants assigned to the subordinate role, higher social vitality was associated with higher negative affect, whereas higher social vitality was associated with lower negative affect among participants assigned to the leader role. A similar cross-over pattern emerged for self-reported role comfort: for participants assigned to the subordinate role, higher social vitality was associated with lower role comfort, whereas higher social vitality was associated with higher role comfort among participants assigned to the leader role.

Table S2.

*Multiple regression models of role, psychopathy (PPI) subscales, and their interactions on negative affect, positive affect, role comfort and psychophysiological reactivity variables.*

|  |  | Psychopathy | | |
| --- | --- | --- | --- | --- |
|  |  | *β* | 95% CI | *p* |
| Negative Affect |  |  |  |  |
|  | Pre-Task Negative Affect | .07 | -.17, .32 | .55 |
|  | Assigned Role | -.02 | -.49, .44 | .96 |
|  | Fearless Dominance | .06 | -.28, .40 | .73 |
|  | Self-Centered Impulsivity | -.06 | -.40, .27 | .70 |
|  | Coldheartedness | -.26 | -.60, .07 | .12 |
|  | Role x Fearless Dominance | -.19 | -.69, .32 | .46 |
|  | Role x Self-Centered Impulsivity | .40 | -.10, .90 | .12 |
|  | Role x Coldheartedness | -.00 | -.49, .49 | .98 |
| Positive Affect |  |  |  |  |
|  | Pre-Task Positive Affect | -.04 | -.27, .19 | .71 |
|  | Assigned Role | .27 | -.16, .70 | .23 |
|  | Fearless Dominance | **-.52** | **-.83, -.21** | **.001** |
|  | Self-Centered Impulsivity | .01 | -.29, .32 | .93 |
|  | Coldheartedness | -.27 | -.58, .04 | .08 |
|  | Role x Fearless Dominance | -.22 | -.69, .25 | .35 |
|  | Role x Self-Centered Impulsivity | -.21 | -.67, .25 | .37 |
|  | Role x Coldheartedness | .02 | -.43, .48 | .92 |
| Role Comfort |  |  |  |  |
|  | Assigned Role | .08 | -.40, .55 | .75 |
|  | Fearless Dominance | .12 | -.22, .46 | .49 |
|  | Self-Centered Impulsivity | -.17 | -.50, .17 | .32 |
|  | Coldheartedness | .04 | -.29, .38 | .80 |
|  | Role x Fearless Dominance | .13 | -.38, .63 | .62 |
|  | Role x Self-Centered Impulsivity | .08 | -.43, .58 | .76 |
|  | Role x Coldheartedness | .01 | -.49, .51 | .96 |
| SCL fPCA2 |  |  |  |  |
|  | Assigned Role | -.17 | -.66, .33 | .53 |
|  | Fearless Dominance | .03 | -.32, .38 | .88 |
|  | Self-Centered Impulsivity | -.21 | -.54, .12 | .21 |
|  | Coldheartedness | .23 | -.14, .59 | .22 |
|  | Role x Fearless Dominance | .48 | -.06, 1.01 | .08 |
|  | Role x Self-Centered Impulsivity | .21 | -.34, .76 | .45 |
|  | Role x Coldheartedness | -.36 | -.88, .16 | .17 |
| RSA fPCA2 |  |  |  |  |
|  | Assigned Role | .41 | -.04, .86 | .08 |
|  | Fearless Dominance | .06 | -.26, .38 | .72 |
|  | Self-Centered Impulsivity | .21 | -.11, .52 | .20 |
|  | Coldheartedness | -.15 | -.49, .19 | .39 |
|  | Role x Fearless Dominance | -.35 | -.84, .13 | .15 |
|  | Role x Self-Centered Impulsivity | .42 | -.06, .89 | .08 |
|  | Role x Coldheartedness | .23 | -.25, .70 | .34 |
| RSA fPCA3 |  |  |  |  |
|  | Assigned Role | .05 | -.44, .44 | .84 |
|  | Fearless Dominance | .30 | -.06, .65 | .10 |
|  | Self-Centered Impulsivity | .10 | -.25, .45 | .58 |
|  | Coldheartedness | .14 | -.23, .52 | .45 |
|  | Role x Fearless Dominance | .20 | -.33, .73 | .45 |
|  | Role x Self-Centered Impulsivity | .08 | -.44, .60 | .76 |
|  | Role x Coldheartedness | -.08 | -.60, .45 | .77 |

*Note. 95% confidence intervals were bootstrapped to increase robustness against violations of multivariate distributional assumptions in the context of small samples.*

Table S3.

*Multiple regression models of role, mania risk (HPS) subscales, and their interactions on negative affect, positive affect, role comfort and psychophysiological reactivity variables.*

|  |  | Mania Risk | | |
| --- | --- | --- | --- | --- |
|  |  | *β* | 95% CI | *p* |
| Negative Affect |  |  |  |  |
|  | Pre-Task Negative Affect | .04 | -.24, .32 | .79 |
|  | Assigned Role | -.06 | -.60, .48 | .31 |
|  | Social Vitality | -.34 | -.73, .05 | .09 |
|  | Mood Volatility | .23 | -.23, .68 | .32 |
|  | Excitement | .39 | -.08, .87 | .10 |
|  | Role x Social Vitality | **.59** | **.00, 1.19** | **.05** |
|  | Role x Mood Volatility | .05 | -.76, .86 | .90 |
|  | Role x Excitement | -.64 | -1.45, .17 | .12 |
| Positive Affect |  |  |  |  |
|  | Pre-Task Positive Affect | -.18 | -.43, .07 | .15 |
|  | Assigned Role | .13 | -.38, .64 | .56 |
|  | Social Vitality | -.09 | -.48, .30 | .65 |
|  | Mood Volatility | .11 | -.33, .55 | .61 |
|  | Excitement | .27 | -.18, .71 | .23 |
|  | Role x Social Vitality | -.25 | -.82, .33 | .39 |
|  | Role x Mood Volatility | .24 | -.53, 1.01 | .54 |
|  | Role x Excitement | -.25 | -1.04, .53 | .52 |
| Role Comfort |  |  |  |  |
|  | Assigned Role | .17 | -.35, .69 | .51 |
|  | Social Vitality | .20 | -.19, .59 | .30 |
|  | Mood Volatility | -.04 | -.49, .41 | .85 |
|  | Excitement | .25 | -.21, .70 | .28 |
|  | Role x Social Vitality | **-.62** | **-1.20, -.03** | **.04** |
|  | Role x Mood Volatility | .02 | -.77, .82 | .95 |
|  | Role x Excitement | -.07 | -.87, .74 | .87 |
| SCL fPCA2 |  |  |  |  |
|  | Assigned Role | -.33 | -.91, .25 | .26 |
|  | Social Vitality | -.17 | -.59, .25 | .43 |
|  | Mood Volatility | -.17 | -.67, .32 | .48 |
|  | Excitement | .16 | -.36, .68 | .53 |
|  | Role x Social Vitality | .45 | -.20, 1.10 | .17 |
|  | Role x Mood Volatility | -.37 | -1.23, .49 | .39 |
|  | Role x Excitement | .23 | -.68, 1.13 | .62 |
| RSA fPCA2 |  |  |  |  |
|  | Assigned Role | .47 | -.03, .96 | .07 |
|  | Social Vitality | .04 | -.35, .42 | .85 |
|  | Mood Volatility | .03 | -.45, .51 | .90 |
|  | Excitement | .04 | -.42, .51 | .85 |
|  | Role x Social Vitality | .14 | -.43, .71 | .62 |
|  | Role x Mood Volatility | **.90** | **.21, 1.58** | **.01** |
|  | Role x Excitement | **-.95** | **-1.66, -0.23** | **.01** |
| RSA fPCA3 |  |  |  |  |
|  | Assigned Role | .47 | -.06, 1.01 | .08 |
|  | Social Vitality | .09 | -.33, .50 | .68 |
|  | Mood Volatility | -.20 | -.71, .31 | .44 |
|  | Excitement | -.30 | -.80, .20 | .24 |
|  | Role x Social Vitality | .11 | -.50, .72 | .72 |
|  | Role x Mood Volatility | .56 | -.18, 1.29 | .13 |
|  | Role x Excitement | -.18 | -.95, .59 | .64 |

*Note. 95% confidence intervals were bootstrapped to increase robustness against violations of multivariate distributional assumptions in the context of small samples.*

In contrast to the self-report outcomes, the Excitement and Mood Volatility subscales of the HPS were associated significantly with fPCA2RSA psychophysiological indices. An interaction between assigned role and Excitement was observed for fPCA2_RSA_, reflecting sustained response from the introduction of the role assignment through the dyadic interaction. For participants assigned to the leader role, higher Excitement scores were related to lower RSA during the dyadic task, consistent with greater engagement of the sympathetic nervous system; in contrast, no association between Excitement and fPCA2_RSA_ was observed for participants assigned to the subordinate role. Conversely, for participants assigned to the leader role, higher Mood Volatility was associated with *higher* fPCA2_RSA_ scores—as with Excitement, however, no association was observed for participants assigned to the subordinate role.

*Psychophysiological PCA factor scores.* Table S4 provides two separate, parallel regression models for the two psychophysiological factor scores that captured tonic levels-- PCA1_SCL_ and PCA1_RSA_. As shown, a main effect of depression on fPCA1_SCL_ was observed such that higher depression scores predicted diminished SCL. This effect was qualified by a significant interaction between depression and assigned role such that greater depression severity related to diminished SCL for participants who were assigned to the subordinate role (*b* = -.71, 95% CI = [-1.21, -.20]), but not for those assigned to the leader role (*b* = -.16, 95% CI = [-.59, .27]), as shown in Figure S3. No other interactions of psychopathology x assigned role were observed for these tonic psychophysiology indices.

Table S4.

*Multiple regression models of role, psychopathy scores, and their interactions on fPCA1_SCL_ and fPCA1_RSA_.*

|  |  | *β* | 95% CI | *p* |
| --- | --- | --- | --- | --- |
| SCL fPCA1 |  |  |  |  |
|  | Assigned Role | -.17 | -.84, .09 | .24 |
|  | Mania Composite | .30 | -.51, .77 | .16 |
|  | Fearless Dominance | .13 | -.25, .62 | .64 |
|  | Depression Composite | **-.67** | **-1.37, -.25** | **.003** |
|  | Social Anxiety Composite | .24 | -.41, .77 | .34 |
|  | Role x Mania | -.25 | -.91, .55 | .23 |
|  | Role x Fearless Dominance | -.02 | -.69, .49 | .93 |
|  | Role x Depression | **.42** | **.04, 1.21** | **.05** |
|  | Role x Social Anxiety | -.06 | -.85, .59 | .80 |
| RSA fPCA1 |  |  |  |  |
|  | Assigned Role | .03 | -.45, .65 | .84 |
|  | Mania Composite | -.03 | -.61, .57 | .90 |
|  | Fearless Dominance | -.06 | -.73, .71 | .84 |
|  | Depression Composite | .32 | -.08, .86 | .23 |
|  | Social Anxiety Composite | -.19 | -.88, .54 | .48 |
|  | Role x Mania | .24 | -.29, .99 | .31 |
|  | Role x Fearless Dominance | .13 | -.68, .94 | .63 |
|  | Role x Depression | -.34 | -.96, .07 | .21 |
|  | Role x Social Anxiety | .17 | -.67, 1.10 | .49 |

*Note. 95% confidence intervals were bootstrapped to increase robustness against violations of multivariate distributional assumptions in the context of small samples.*

**Figure S3**

*Interaction between assigned role and depression symptoms predicts skin conductance (SCR PCA1) patterns.*

**Supplementary Discussion.** Overall, supplementary analyses yielded few statistically significant findings. This is particularly unsurprising for the analyses exploring gender, as the three-way interactions lack adequate statistical power.

Consistent with theory, analyses of subscales of the PPI did not indicate that factors other than Fearless Dominance were tied to social dominance variables. Together with findings reported in the main body of the paper, we saw little evidence that any of the psychopathy (PPI) scales were related to reactivity to the assigned role.

A potentially more intriguing and interpretable exploratory finding is that different factors of the HPS appeared to track specificfacets of reactivity to the social interaction. Social Vitality scores predicted self-reported post-task negative affect and role comfort, which would indicate that the dominance effects observed for the HPS may be particularly related to the Social Vitality aspects of mania risk, which is consistent with the focus of these items on interpersonal function. In contrast, the Excitement scores predicted diminished RSA activity, consistent with the Excitement subscale focus on high arousal energetic states.

Although we had no a priori hypotheses regarding tonic psychophysiology indices, depression severity was related to lower SCL scores throughout the recording period for those assigned to the subordinate role.

**Supplementary References**

1. Berg JM, Hecht LK, Latzman RD, Lilienfeld SO. Examining the correlates of the coldheartedness factor of the Psychopathic Personality Inventory–Revised. *Psychological Assessment 2015 April; 27*(4): 1494–1499. [https://doi.org/10.1037/pas0000129](https://psycnet.apa.org/doi/10.1037/pas0000129)
2. Schalet BD, Durbin CE, Revelle W. Multidimensional structure of the Hypomanic Personality Scale. *Psychological Assessment 2011 Feb; 23*(2): 504–522. [https://doi.org/10.1037/a0022301](https://psycnet.apa.org/doi/10.1037/a0022301)


Experimenter Instructions for the Dyadic Task (***Participant as leader*)**

- After baseline, return to main room with confed. Bring blue binder and a blank dyad score sheet.
- *Stay standing,* [say partici name] *you can remain on that end of the table and* [say confed’s name] *you can stay at this end* [indicate the end closest to the door]
- Tell them both: *Now, for the next task, I need to assign you to either a leader or subordinate role. Remember you filled out some online questionnaires earlier in the session?* (look to P and confed for some head nodding)*.*
- *Well, I made your role assignments based on your responses to some of these earlier questionnaires.* Always start by announcing participant’s role
  - [say partici name]*You have been assigned as the leader. As leader, you get to sit in this blue chair.* [While putting $7 on the table in front of the leader to distribute]  *I will explain the purpose of the money in a minute.*
  - [say confed’s name]*You have been assigned to the subordinate role. As the subordinate, you have to sit in this gray chair.*
- *Now let me explain the task and your roles.*
- Place blue binder and blank score sheet on the table. We will be coding for if the participant reaches for pen/turns pages/etc so it should be set up equidistance from them; confeds in leader role are told to reach first, but don’t say anything if either reaches before you finished
- *The pictures in this binder are the ones you just rated. I went ahead and compared the ratings you each made individually. As we mentioned, the ability to make accurate personality judgments is an important skill.*
- *There were some discrepancies in your ratings for quite a few pictures, but we’d like you to focus on these 4 with the greatest discrepancy* [highlight the trait box of the corresponding picture on the blank score sheet]*. Discussing these with another person will help us understand your rationale for choosing the ratings, and allow us to understand the process of how people in pairs make decisions and judgments together. Here are your original score sheets* [hand them their individual score sheets: subordinates on a clipboard, and place leaders on desk].
- *For each rating that I have circled, we would like you to state your rating of the picture and then* ***describe the rationale*** *behind your judgment. So, for example, if you decide to give a very high rating of one of the photos on the trait dimension of extraversion, you might justify this rating by the fact that the person’s expression is very natural and inviting.*
- *After you have each stated your ratings and rationales, we’d like you to* ***discuss*** *any discrepancy and decide on who made the more accurate rating, and* ***enter a final single*** *rating on the score sheet.*
- Always start by announcing participant’s role, then confed’s.
  - [Partici name] a*s leader, you get to state your ratings and rationales first and then* [confed’s name] *will have to say theirs.* [Partici name] a*s leader, you have a $7 paycheck. You can decide to divide this paycheck between yourself and your partner should you choose to at the end of the session. But please do not make your decision about how to distribute the paycheck until my instructions.*
- *Please note that we are as interested in your process of coming to a decision on each personality rating, as we are in the ratings themselves, so we ask you to* ***truly engage*** *in conversation. You will have 15 minutes to convey your ratings and rationale, and come to a decision for the 4 ratings. We will let you know when there are 5 minutes left. As a reminder, we are both video and audio recording you during this task. Remember to discuss only the 4 ratings I’ve circled on the score sheet. Do you have any questions?*
